# A case report of distal coronary artery spasm overlapping a calcified nodule: a rare mechanism of acute coronary syndrome

**DOI:** 10.1093/ehjcr/ytag269

**Published:** 2026-04-15

**Authors:** Takuya Matsumoto, Hiroyuki Kiriyama, Takashi Hiruma, Shun Minatsuki, Norihiko Takeda

**Affiliations:** Department of Cardiovascular Medicine, The University of Tokyo, 7-3-1 Hongo, Bunkyo-ku, Tokyo, 113-8655, Japan; Department of Cardiovascular Medicine, The University of Tokyo, 7-3-1 Hongo, Bunkyo-ku, Tokyo, 113-8655, Japan; Department of Cardiovascular Medicine, The University of Tokyo, 7-3-1 Hongo, Bunkyo-ku, Tokyo, 113-8655, Japan; Department of Cardiovascular Medicine, The University of Tokyo, 7-3-1 Hongo, Bunkyo-ku, Tokyo, 113-8655, Japan; Department of Cardiovascular Medicine, The University of Tokyo, 7-3-1 Hongo, Bunkyo-ku, Tokyo, 113-8655, Japan

**Keywords:** coronary artery spasm, Calcified nodule, Acute coronary syndrome, Case report

## Abstract

**Background:**

Calcified nodules (CNs) are the least common cause of acute coronary syndrome (ACS) and are distinct from plaque rupture and erosion. Although CNs are increasingly recognized as a unique ACS mechanism, their pathophysiology remains poorly understood.

**Case summary:**

A 66-year-old man with a long history of alcohol-related chest discomfort was referred for evaluation of worsening exertional symptoms. The initial electrocardiogram was unremarkable, but coronary computed tomography angiography showed a moderately calcified stenosis in the mid–right coronary artery (RCA), and elective coronary angiography was scheduled. However, on admission for the planned procedure, the patient experienced chest discomfort following binge drinking the night before. The electrocardiogram on admission revealed inferior ST-segment elevation. Emergent coronary angiography and intracoronary imaging identified severe mid–RCA stenosis associated with a CN with an overlying thrombus, while distal flow was preserved (Thrombolysis in Myocardial Infarction grade 3). The subsequent acetylcholine provocation test was positive, confirming coronary spastic angina (CSA). These findings raise the possibility that distal embolization from the surface thrombus on the CN contributed to the ACS presentation, potentially facilitated by CSA-associated flow reduction.

**Discussion:**

This case highlights a potential dual mechanism of ACS, in which distal coronary artery spasm may have promoted thrombus formation on a CN by reducing coronary blood flow. The coexistence of CN and CSA may represent a synergistic pathophysiology linking mechanical plaque vulnerability with flow-mediated thrombus formation.

Learning points

- Distal coronary artery spasm may contribute to CN-related ACS by reducing coronary blood flow and potentially promoting thrombus formation on irregular calcified surfaces.
- Detailed history taking and precise intracoronary imaging evaluation can enable appropriate assessment of the mechanism and guide treatment.

## Introduction

Calcified nodules (CNs) are the least common aetiology of acute coronary syndrome (ACS), following plaque rupture and plaque erosion.^1^ Although CNs have gained attention as an emerging concept in ACS, their pathophysiology remains poorly understood. Here, we report a case of recurrent distal coronary artery spasms that potentially contributed to CN-related ACS.

## Summary figure

**Figure:**
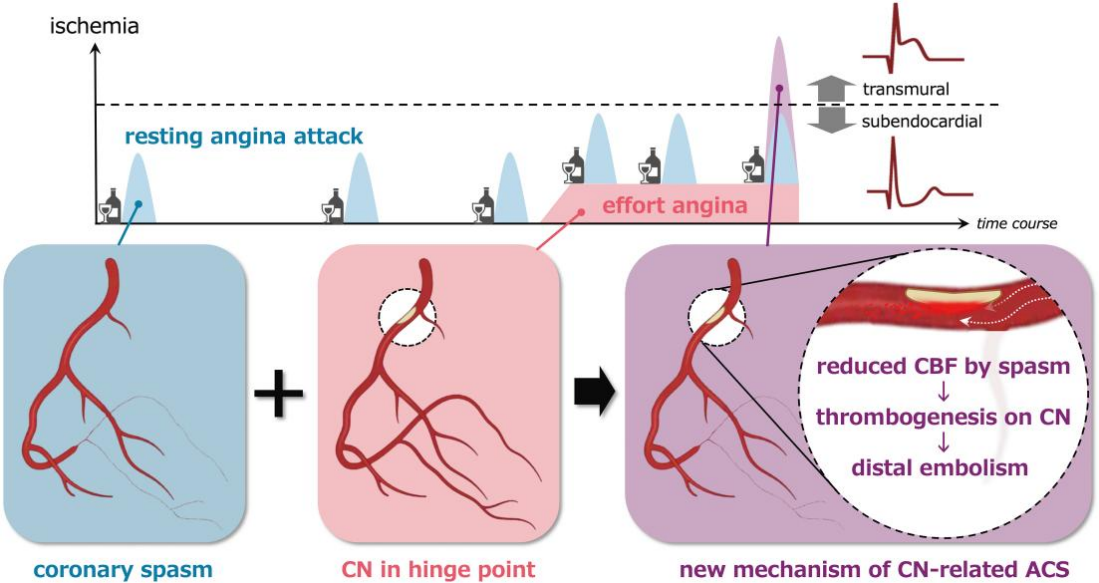
(Proposed mechanism). ACS, acute coronary syndrome; CBF, coronary blood flow; CN, calcified nodule.

## Timeline

CAG, coronary angiography; CN, calcified nodule; CSA, coronary spastic angina; DAPT, dual antiplatelet therapy; PCI, percutaneous coronary intervention; RCA, right coronary artery.

**Figure:**
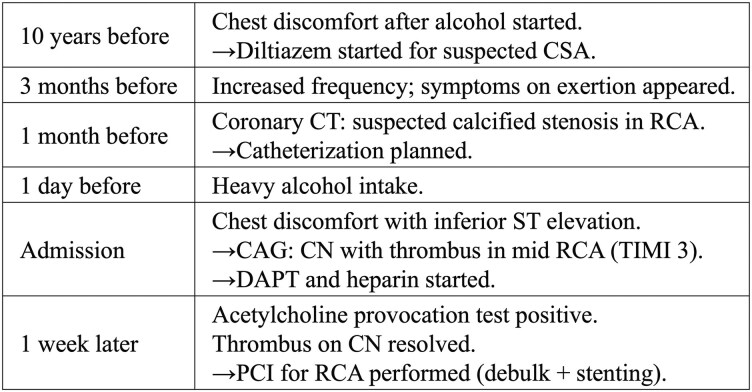


## Case presentation

**Figure 1:**
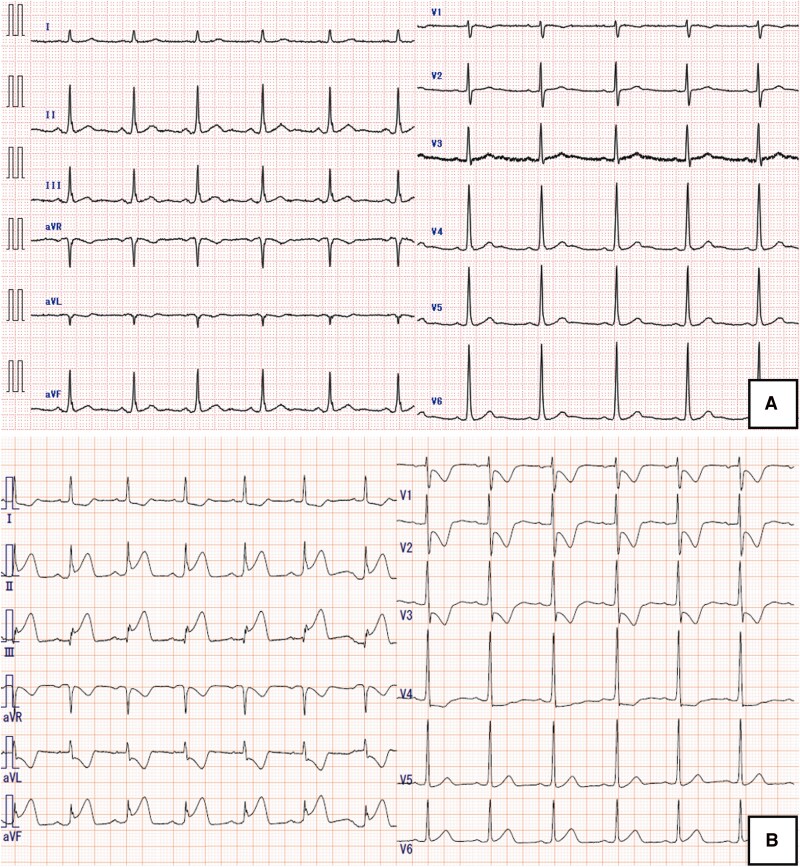
(*A*) Baseline electrocardiogram at initial outpatient evaluation showing normal results. (*B*) Electrocardiogram at the index ACS presentation showing ST-segment elevation in the inferior leads.

A 66-year-old man presented with a long-standing history of chest discomfort occurring early in the morning and after alcohol consumption. Despite ongoing treatment with diltiazem for suspected coronary spastic angina (CSA), his symptoms became more frequent and progressed to exertional angina, prompting referral to our hospital. He had a history of dyslipidaemia (low-density lipoprotein cholesterol 149 mg/dL) and was a former smoker (12 pack-years). Troponin level, electrocardiogram (ECG), and echocardiography were unremarkable at the initial outpatient evaluation (*Figure 1A*). Coronary computed tomography angiography revealed moderately calcified stenoses in the mid–right coronary artery (RCA) and left anterior descending (LAD) artery. Based on these findings, elective coronary angiography was scheduled.

**Figure 2:**
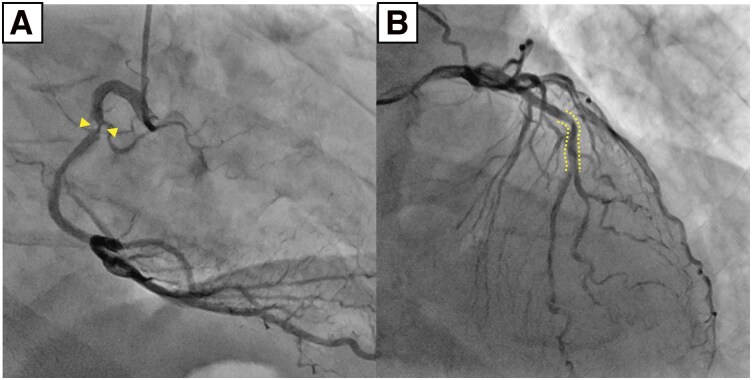
Initial coronary angiography showing severe stenoses in the mid–right coronary artery (arrowheads in *A*) and in the left anterior descending artery (dotted lines in *B*).

**Figure 3:**
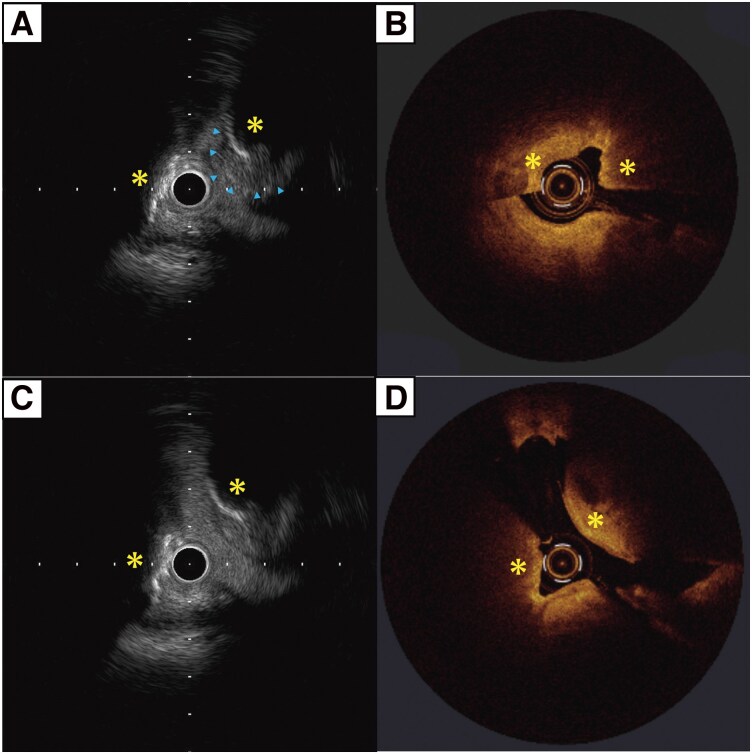
(*A*, *B*) Index ACS event: intravascular ultrasound (IVUS) and optical coherence tomography (OCT) images demonstrating calcified nodules (CNs) (yellow asterisks) with overlying thrombus (blue arrowheads). (*C*, *D*) Follow-up (one week after intensive antithrombotic therapy): repeat IVUS and OCT at the same site, showing persistent CNs (yellow asterisks) with thrombus resolution.

On admission for the procedure, the patient complained of chest discomfort after binge drinking the night before. The ECG showed ST-segment elevation in the inferior leads (*Figure 1B*), and emergent cardiac catheterization was performed. Coronary angiography revealed severe stenosis in the mid–RCA and LAD (*Figure 2*) with preserved distal Thrombolysis in Myocardial Infarction grade 3 (TIMI 3) flow. The ECG findings implicated the RCA as the culprit vessel, and intracoronary imaging of the mid-RCA revealed a CN with an overlying thrombus and no evidence of plaque rupture or erosion (*Figure 3A* and *B*). Taken together, these findings raised suspicion that distal embolization from the surface thrombus may have contributed to the persistent ST-segment elevation and chest discomfort. As the patient was haemodynamically stable with preserved TIMI 3 flow and had no ongoing chest pain, we deferred immediate stenting to avoid further embolization and prioritized intensive antithrombotic therapy (dual antiplatelet therapy and heparin), planning staged PCI thereafter. We also scheduled an acetylcholine provocation test at the beginning of the staged procedure to confirm vasospastic angina and to guide long-term vasodilator therapy.

**Figure 4:**
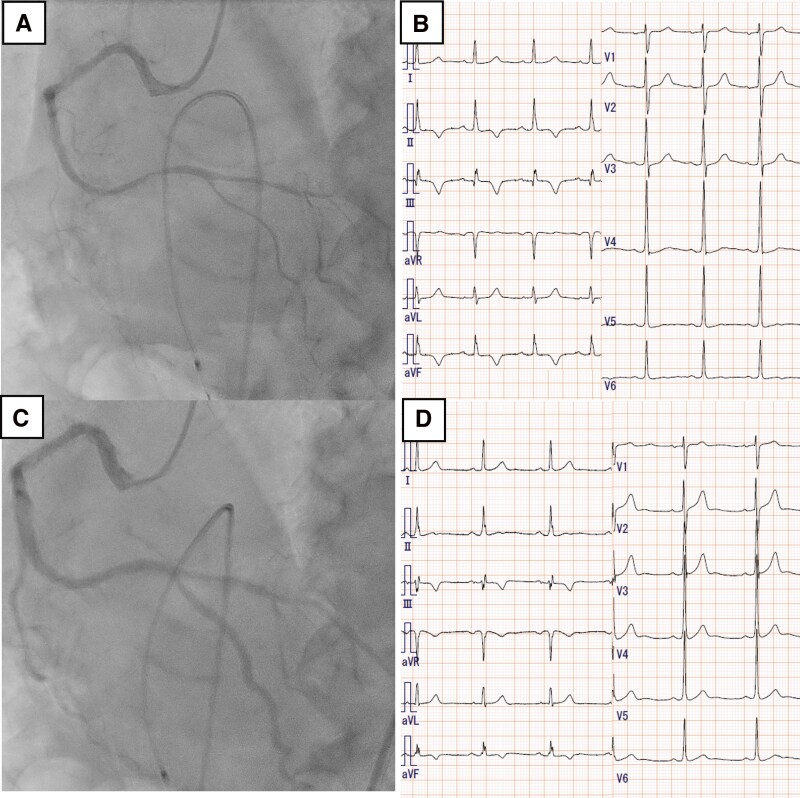
Acetylcholine provocation test showing diffuse >90% narrowing in the distal right coronary artery branches (*A*), accompanied by chest discomfort and inferior ST-segment depression (*B*). Intracoronary injection of nitroglycerin relieved the stenosis in the distal right coronary artery (*C*) and improved chest discomfort and the electrocardiogram changes (*D*).

One week later, PCI was performed for the mid–RCA stenosis. The initial acetylcholine provocation test revealed >90% narrowing in the distal RCA branches accompanied by typical chest discomfort and inferior ST-segment depression, confirming CSA (*Figure 4*). Follow-up intracoronary imaging showed complete resolution of the thrombus and a smooth, non-eruptive CN (*Figure 3C* and *D*). These findings are consistent with the hypothesis that recurrent distal coronary artery spasms may have reduced coronary blood flow and promoted stasis-related thrombus formation on the CN, rather than nodule eruption. Subsequently, rotational atherectomy was performed for lesion preparation, followed by implantation of a drug-eluting stent. The patient remained free from recurrent chest pain after further intervention for the LAD lesion, reduced alcohol intake, and initiation of nicorandil.

## Discussion

This case illustrates ACS with coexisting CSA and a CN. The patient’s long-standing early-morning and alcohol-related chest discomfort is most consistent with recurrent CSA, supported by a positive acetylcholine provocation test. In contrast, the recent onset of exertional angina likely reflected flow-limiting organic stenosis at the mid–RCA lesion with a CN.

A CN is recognized as the third mechanism of ACS, distinct from plaque rupture and erosion. It is defined as a crater-like nodular calcification that protrudes into the coronary artery lumen, and CN-related ACS is typically accompanied by overlying thrombus formation. Although the mechanisms of CN formation remain unclear, one hypothesis is that mechanical stress fragments calcium sheets, resulting in small nodules. These nodules may eventually disrupt the overlying fibrous cap and endothelial lining, causing luminal thrombosis (so-called eruptive CN). CNs tend to localize in the mid–RCA, as in this case, where excessive torsion or hinge motion during the cardiac cycle is pronounced.^1–3^

During the ACS event, the patient developed persistent inferior ST-segment elevation with chest discomfort despite preserved TIMI 3 flow. Several explanations could be considered. Severe epicardial and/or microvascular spasm causing transient occlusion was one possibility.^4^ However, the acetylcholine provocation test did not reproduce epicardial occlusion or ST-segment elevation, so spasm alone was less likely to explain the presentation. Myocardial stunning after a transient occlusive event was also considered, but intracoronary imaging showed no features of plaque rupture or erosion, which made a classic thrombotic occlusion with spontaneous reperfusion less convincing. Given these considerations and the presence of an overlying thrombus on the CN surface, distal embolization from the surface thrombus was suspected as a plausible leading explanation for ST-segment elevation with chest discomfort.

A further key observation was obtained after intensive antithrombotic therapy. Follow-up intracoronary imaging showed complete thrombus resolution and a smooth, non-eruptive CN. This appearance argues against a typical eruptive CN mechanism in which disruption of the overlying surface directly drives thrombosis. Instead, we considered a spasm-related low-flow milieu as a potential contributor to thrombus formation on the CN. Distal epicardial or microvascular spasm can markedly reduce coronary flow.^5,6^ Reduced coronary blood flow is also known to promote thrombosis on irregular surfaces, including plaques and CNs.^7,8^ In this patient, the long history of alcohol-triggered CSA and the occurrence of ACS the day after binge drinking support recurrent spasm as a potential trigger. Taken together, recurrent distal spasm may have created a low-flow milieu, facilitated thrombus formation on the smooth non-eruptive CN surface, and led to intermittent distal embolization, thereby producing transmural ischaemia despite preserved TIMI 3 flow.

Recognizing this dual mechanism has practical implications. In patients with CN-related lesions and concomitant CSA, management may require both structural treatment of the stenosis and spasm-directed therapy to prevent recurrent spasm and low-flow states. In the present case, deferring immediate stenting to prioritize thrombus resolution, followed by staged PCI and vasodilator therapy, provided a coherent strategy consistent with the suspected pathophysiology.

Our case presentation has some limitations. First, conventional mechanical factors in the mid–RCA, such as torsion, hinge motion, and focal shear stress, likely contributed to CN and thrombus formation and cannot be excluded as primary drivers. However, these mechanisms alone do not readily explain the concomitant distal provoked spasm, the persistent ST-segment elevation despite preserved TIMI 3 flow, or the non-eruptive CN appearance after thrombus resolution. Second, our proposed sequence—alcohol-triggered spasm, a low-flow state, thrombus formation on the CN, and distal embolization—is inferential and based on indirect evidence. Third, the acetylcholine provocation test was performed one week after the index ACS event; coronary vasomotor responses can be altered early after ACS,^9^ and the results should therefore be interpreted cautiously.

In conclusion, recurrent distal coronary artery spasms may contribute to CN-related ACS through persistent reduction in coronary blood flow and increased thrombogenicity on irregular calcified surfaces. Recognizing the coexistence of spasms and CN may help in understanding ACS mechanisms and provide a rationale for patient management. Clinically, detailed history taking and precise imaging evaluation enable appropriate assessment of the pathological condition and optimal treatment selection.

## Lead author biography


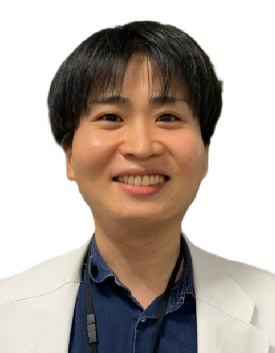


Takuya Matsumoto is a cardiology trainee at the University of Tokyo Hospital, Japan. His interest is focused on coronary physiology, hemodynamics, and heart failure.

## Data Availability

All data relevant to this case report are included in this article and its figures. Additional anonymized details may be made available by the corresponding author upon reasonable request, subject to patient privacy and institutional requirements.

## Patient perspective

A patient perspective statement was not obtained.
